# Health Benefits, Antioxidant Activity, and Sensory Attributes of Selected Cold-Pressed Oils

**DOI:** 10.3390/molecules28145484

**Published:** 2023-07-18

**Authors:** Dobrochna Rabiej-Kozioł, Monika Momot-Ruppert, Barbara Stawicka, Aleksandra Szydłowska-Czerniak

**Affiliations:** 1 Department of Analytical Chemistry and Applied Spectroscopy, Faculty of Chemistry, Nicolaus Copernicus University in Toruń, Gagarina 7, 87-100 Toruń, Poland; d.rabiej@umk.pl (D.R.-K.); monika.momot@bunge.com (M.M.-R.); 2 Bunge Polska Sp. z o.o., Niepodległości 42, 88-150 Kruszwica, Poland; barbara.stawicka@bunge.com

**Keywords:** cold-pressed oils, antioxidants, antioxidant activity, nutritional indicates, oxidative stability, hazardous compounds, sensory analysis

## Abstract

The consumption of cold-pressed oils (CPOs) has continuously increased due to their health-promoting compounds, such as polyunsaturated fatty acid (PUFA), tocopherols, sterols, and polyphenols. This study focused on the estimation and comparison of the physicochemical properties and sensory quality of six CPOs: linseed oil (CPLO), pumpkin oil (CPPO), milk thistle oil (CPMTO), rapeseed oil (CPRO), camelina oil (CPCO), and sunflower oil (CPSO), which are the most popular in the Polish market. These oils were analysed for their fatty acid composition (FAC), their tocopherol, sterol, polycyclic aromatic hydrocarbon (PAHs), water, and volatile matter (WVM) contents, as well as their antioxidant activity (AA) and oxidative stability parameters. Moreover, quantitative descriptive analysis (QDA) was performed to obtain detailed information on the sensory profiles and quantitative data on the CPOs’ attributes that affected consumer acceptability and purchase intent. All of the analysed CPOs were rich in PUFA (27.94–68.42%). They were characterised by the different total amounts of health-beneficial compounds, such as tocopherols (TTC = 44.04–76.98 mg/100 g), sterols (TSC = 300–684 mg/100 g), and polyphenols (TPC = 2.93–8.32 mg GA/100 g). Additionally, their AA was determined using 2,2-diphenyl-1-picrylhydrazyl (DPPH), 2,2′-azino-bis(3-ethylbenzothiazoline-6-sulfonic acid) (ABTS), and ferric reducing antioxidant power (FRAP) methods, with results ranging between 185.36–396.63, 958.59–1638.58, and 61.93–119.21 µmol TE/100 g, respectively. However, the deterioration parameters of CPOs, such as peroxide values (PV = 0.24–4.61 meq O_2_/kg), p-anisidine values (pAnV = 0.39–4.77), acid values (AV = 0.31–2.82 mg KOH/g), and impurity amounts (Σ4PAHs = 1.16–8.76 μg/kg and WVM = 0.020–0.090%), did not exceed the level recommended by the Codex Alimentarius Commission. The obtained results indicated that all of the investigated CPOs are valuable sources of health-promoting bioactive compounds.

## 1. Introduction

In recent years, cold-pressed oils (CPOs) have received increased attention due to their health-beneficial impact. The advantages of these oils over refined ones are related to higher amounts of bioactive substances, not being otherwise removed during refining processes. Another reason for the growing popularity of CPOs over refined oils is consumers choosing fewer processed products. According to the Codex Alimentarius [1], the CPOs have to be produced only by mechanical procedure, without heat treatment. These oils can be purified by washing them with water, then settling, filtering, and centrifuging them. Chemical and physical refining processes, such as degumming, neutralisation, bleaching, and deodorisation, as well as supplementation of CPOs with synthetic additives, are banned [2,3]. For this reason, CPOs are rich sources of minor constituents, such as the phytosterols, tocopherols, carotenoids, and polyphenols that are associated with health benefits. These oils are also distinguished by their high amounts of vitamins and minerals.

Additionally, CPOs have characteristic flavours and aromas, depending on the seeds or fruits from which they were pressed [4,5]. The characteristic aroma and taste has led to CPOs being mainly used as salad dressing, spread, or otherwise consumed directly. In addition, some CPOs containing high content of oleic acid can be applied for culinary operations at higher temperatures, such as for frying and cooking [6,7].

Furthermore, each CPO has a characteristic fatty acid composition (FAC) and profiles of accompanying compounds, such as tocopherols, sterols, and polyphenols. The unique minor compounds and FAC have been linked with the health-promoting attributes of these oils [4]. Cold-pressed camelina oil (CPCO) can be helpful for skin ailments, cardiovascular diseases, cancer, and chronic diseases [8]. Similarly, cold-pressed milk thistle oil (CPMTO) can be used for skin ailments, such as pruritic, psoriatic, or exsiccated skin, as well as facilitate the treatment of liver- and bile-related disorders due to its anti-inflammatory properties [9]. Additionally, CPMTO reduces paracetamol hepatotoxicity [4]. Cold-pressed linseed oil (CPLO) has preventive properties for coronary heart disease, some types of cancer, and neurological and hormonal disorders [10]. Cold-pressed pumpkin oil (CPPO) has become an effective means for the treatment of both cardiovascular diseases during menopause and benign prostatic hyperplasia in men, as well as androgenic alopecia [11]. Also, cold-pressed sunflower oil (CPSO) shows cardiovascular benefits associated with reduced total plasma cholesterol and low-density lipoprotein (LDL) cholesterol levels. In addition, this oil is helpful in lowering atherosclerosis, artery disease, and stroke, due to the presence of vitamin E at high levels [12]. However, the health-benefit properties of cold-pressed rapeseed oil (CPRO) are linked with regulating blood lipid profile, insulin sensitivity, and glycaemic control [13].

The health-promoting and sensory properties of CPOs have led to some oils being considered new functional and luxury products [14]. Especially niche CPOs should have an evident colour, clarity, and a fresh and clean aroma characteristic of the oil plants. However, it is well known that the origins of oilseeds and various cultivars, as well as their processing techniques, are associated with the composition of minor compounds and the organoleptic characteristics of oils. Therefore, previous studies have demonstrated that there have been differences in the overall quality, including the physicochemical and sensory properties, of commercially available CPOs derived from black cumin seeds, camelina seeds, rapeseed, and sunflower seeds [2,8,15,16]. Nevertheless, a sensory analysis allows the estimation of the proposed oil products’ quality, freshness, and consumer acceptability. In recent studies, quantitative descriptive analysis (QDA) has been a well-known and essential tool for quantifying the sensory attributes of oils that have been cold-pressed from various oilseeds, as well as for facilitating the statistical description of the obtained results [2,8,15].

However, consumers are not willing to compromise on healthful properties or the sensory quality of functional products. Functional compounds present in CPOs, such as phenolics, are often associated with diminished acceptability to consumers, due to their bitter or pungent flavour [17]. Furthermore, one of the most critical factors influencing the sensory quality and nutritional value of CPOs is the oxidation process of unsaturated fatty acids (primarily linolenic acid), as well as the degradation and volatilisation of bioactive compounds. Primary (peroxides) and secondary (aldehydes, ketones, and hydrocarbons) oxidation products contribute to the unpleasant flavours and aromas associated with rancid oils and damage to health, lowering consumer acceptance. The secondary oxidation product, E-2-heptenal, was responsible for the unpleasant rancid or fried odour of sunflower oil, while cyclic octapeptide (consisting of eight amino acids: proline, leucine, phenylalanine, isoleucine, sulfoxidised methionine, leucine, valine, and phenylalanine) caused a bitter ‘off’ taste in oil cold-pressed from linseed [2,18]. Thus, there has been a negative relationship between the perceived healthiness of some oils and fats and their sensory attributes.

On the other hand, because of the lipophilic nature of polycyclic aromatic hydrocarbons (PAHs), unsafe levels of these carcinogenic contaminants can be found in oils and fats. Oil contamination by PAHs may be attributed to the contact of the oilseeds with polluted air, the uptake by the oilseed plants through soil, and any contaminated element present in the production line or mineral oil residues from packaging [19].

Many research studies have demonstrated that the quality control plays an important role in the safety assessment of edible oils obtained by mechanical operations [2,20].

It is worth mentioning that information about the health properties of functional compounds (e.g., plant sterols, liposoluble vitamins, phenolics) in oils influence consumer sensory profiling and purchasing intentions. Hence, extrinsic properties and intrinsic sensory attributes of oils play a crucial role in purchasing intentions, and they can be a fundamental tool for the consumer’s judgement of the oil quality.

To the best of our knowledge, the chemical composition, oxidative stability, and sensory properties of oils pressed from various oilseeds have been studied previously. However, relationships between the antioxidant, nutritional, and healthy features of CPOs and their sensory profiles have not yet been reported.

Therefore, following were the aims of this research: (I) To determine the profiles of fatty acids and liposoluble bioactive compounds (tocopherols and sterols), as well as total phenolic content (TPC) and antioxidant activity (AA) of six CPOs: CPLO, CPPO, CPMTO, CPRO, CPCO, and CPSO, which are the most common in the Polish retail market; (II) To investigate their oxidative status parameters and contaminants; (III) To calculate the nutritional quality indexes of oils based on their FACs for the prevention of cardiovascular diseases; (IV) To develop a vocabulary with adequate descriptors for oils and analysis of their sensory properties and consumers’ acceptance. Moreover, multivariate analyses, such as principal component analysis (PCA) and hierarchical cluster analysis (HCA), were applied to check similarities and differences between the investigated CPOs and physicochemical, nutritional, and sensory properties.

For the first time, these studies have provided additional information to promote healthy oils containing bioactive compounds without undesirable compounds and have presented sensory attributes to create their unique position in the marketplace.

## 2. Results and Discussion

### 2.1. Antioxidant Characteristics of Cold-Pressed Oils

#### 2.1.1. Fatty Acid Compositions and Nutrient Values

The fatty acid profiles have been frequently used to indicate the quality of CPOs, especially for the identification of their authenticity. Each year in the European Union, food frauds are detected where virgin oils have been adulterated with other vegetable oils. As can be seen in Table 1, all of the studied oils had typical fatty acid compositions recommended by Codex Alimentarius standard [1] and similar to those described by other authors [4,5,8,10,16,20].

On the other hand, the fatty acid profile of oil can affect its vulnerability to oxidation and deterioration reactions. Polyunsaturated fatty acids (PUFA) such as linoleic acid (C18:2) and linolenic acid (C18:3) are much more prone to oxidation than monounsaturated (MUFA) oleic acid (C18:1) and saturated (SFA) stearic acid (C18:0) [3,21,22,23].

The total SFA content in the investigated oil samples varied from 7.12 to 19.53%. Among SFA, palmitic (C16:0) and stearic (C18:0) acids were predominantly found in the analysed oils, having percentages of 4.63–12.41% and 1.61–5.90%, respectively. Similar moderate total SFA content in CPLO (11.63%) and CPSO (11.20%) were observed, while CPMTO (19.53%) and CPPO (18.92%) were the richest sources of SFA (Table 1). Moreover, arachidic acid (C20:0 = 2.90%) and behenic acid (C22:0 = 1.91%) reached the highest percentages in CPMTO from all of the evaluated oils. 

It is well known that high SFA intake increases the risk of developing metabolic syndrome, diabetes, cardiovascular diseases, heart failure, and mortality. Therefore, the consumption of oils with high SFA content should be limited in order to maintain a healthy life. Ulbricht and Southgate [24] classified short-chain SFA such as lauric (C12:0), myristic (C14:0), and C16:0 acids as atherogenic, while C14:0, C16:0 and C18:0 as thrombogenic SFA.

For comparison, a similar amounts of total SFA and C16:0 were determined in oils pressed from rapeseed (SFA = 6.73–7.32% and C16:0 = 4.13–4.33%) and pumpkin seeds (SFA = 18.27% and C16:0 = 12.23%) [23,25].

Moreover, CPRO revealed the highest total content of MUFA and the lowest total content of SFA, with values of 64.83% and 7.12%, respectively (Table 1). However, CPLO had the highest PUFA content (68.42%), among the investigated samples. 

Choo et al. [26] reported similar values of C18:3 ranging between 51.8 and 60.4% for linseed oils.

It can be noted that the C18:1 acid (63.26%) was predominant in CPRO, while its percentage in CPSO and CPPO was approximately two times lower (30.64 and 30.40%, respectively) than in CPRO. Among the studied oils, the lowest concentration of C18:1 (18.11–24.69%) was found in CPCO, CPLO, and CPMTO. Interestingly, gadoleic acid (C20:1 = 14.43%) was at the highest level in CPCO. Similar results of C18:1 content (12.01–23.57%) in market linseed oils were reported by Symoniuk et al. [27].

Taking into consideration the health benefits of MUFA, especially C18:1, and their effect on the oxidative stability of the oils, there is a need to select CPOs with high amounts of MUFA. It has been documented that MUFA can reduce LDL cholesterol and increase high-density lipoprotein (HDL) cholesterol levels. 

Generally, in all CPOs (except CPRO), PUFA (49.67–68.42%) dominated (Table 1). It is noteworthy that C18:2 (50.39–57.64%) was the major PUFA in CPPO, CPMTO, and CPSO. However, C18:3 was the most abundant PUFA in CPLO and CPCO (52.12 and 30.29%, respectively). 

Other researchers have presented similar amount of C18:2 in CPPO (44.95–53.59%) and CPMTO (51.78–53.87%) [20,23]. Furthermore, the content of C18:3 in 15 linseed oils ranged between 44.90 and 64.62% [27].

Although CPRO had a relatively low C18:3 level (8.12%), the nutritionally desirable ratio of omega-6 (ω-6) to omega-3 (ω-3) fatty acids (about 2:1) in this oil was observed [13]. The optimal ratio of ω-6:ω-3 fatty acids, close to balancing, is recommended for normal physiological functions in the body, and it is crucial in preventing many chronic diseases such as cancer, inflammatory and autoimmune, and cardiovascular diseases. This fact is of great importance for the regular intake of CPRO and its effect on human health. Nevertheless, the concentration of C18:3 in CPPO, CPMTO, and CPSO did not exceed 0.3% (Table 1).

Some recent studies have found that PUFA in vegetable oils can prevent coronary heart and cardiovascular diseases, cancer, inflammatory, thrombotic and autoimmune diseases, hypertension, type two diabetes, renal diseases, rheumatoid arthritis, ulcerative colitis, and Crohn’s disease [12,13,17].

On the other hand, FACs provide information about the oxidative stability of oils and their nutritional values. In addition, based on the percentage of fatty acids in the studied CPOs, the oxidisability values (COX) associated with oxidative stability, as well as nutritional quality indicators related to cardiovascular and heart diseases, such as atherogenicity index (AI), thrombogenicity index (TI), and the ratio of hypocholesterolemic to hypercholesterolemic (HH) fatty acids, were calculated. These indicators were more helpful for the evaluation of the nutritional quality of oils and their autoxidation rates than the fatty acid profiles [6,28].

It can be noted that the COX values of the studied commercially available CPOs ranged from 4.43 for CPRO to 13.14 for CPLO (Table 1). These COX values increased in the following order: CPRO < CPPO < CPMTO < CPSO < CPCO < CPLO. The calculated COX results proved that CPLO had the highest susceptibility to oxidation, while CPRO was the most stable in autoxidation reactions among the investigated oils. The obtained COX values indicate that there were relationships between the amounts of SFA, MUFA, and PUFA in CPOs and their susceptibility to oxidation.

Generally, the COX values calculated for the six CPOs were similar to those observed by Symoniuk et al. [20,27] for CPLO (12.03–15.40), CPRO (4.27–4.41), CPMTO (5.64–5.86), CPCO (8.76–9.42), although CPPO revealed slightly higher COX indexes (6.07–6.32).

Additionally, levels of SFA, MUFA, and PUFA and the ω-6:ω-3 ratio significantly affect the dietary factors of oils. The AI and TI indices characterise the effects of individual fatty acids present in oils on human health, mainly the incidence of atherosclerosis, the development of blood clots, atheroma, and thrombus formation [29]. From the nutritional point of view, low values of AI and TI are preferred in the human diet. Furthermore, the HH ratio is an index related to cholesterol metabolism. Higher HH ratios are considered to be more beneficial to human health [30]. Hypocholesterolemic acids, such as unsaturated C18 and C20 fatty acids, are more effective in decreasing total cholesterol level, whereas C14:0 and C16:0 are classified as hypercholesterolemic acids increasing cholesterol level.

The AI and TI, amounting to between 0.02–0.08 and 0.06–0.45, respectively, were low for all investigated CPOs (Table 1). The lowest AI (0.02) and the highest HH ratio (19.53) had CPRO. In contrast, the highest values of AI (0.08) and TI (0.45), as well as the lowest HH ratio (6.47) were observed for CPPO. The calculated AI, TI, and HH results indicate that enriching the diet with studied oils can reduce coronary heart diseases.

Similar AI and TI values for rapeseed oil (0.05 and 0.09), sunflower oil (0.06 and 0.18), and linseed oil (0.06 and 0.05) were reported by Khalili Tilami et al. [29], while pumpkin oil revealed higher values of AI (0.21) and TI (0.47) than our results (AI = 0.08 and TI = 0.45).

For comparison, AI, TI, and HH values for refined rapeseed oil were 0.04, 0.09, and 21.91, respectively [6]. However, higher AI (0.23) and TI (0.53), as well as lower HH (4.15) for CPMTO were calculated by Ying et al. [31].

#### 2.1.2. Tocopherol Profiles

Tocopherols play an important role in the antioxidant properties of oils rich in PUFA. These lipophilic antioxidants can chelate metal ions and scavenge free radicals. Thus, they are the most effective in protecting the oil from oxidation processes [4]. As can be seen in Table 2, total tocopherol content (TTC) in the tested CPOs ranged between 44.04 mg/100 g for CPLO and 76.98 mg/100 g for CPCO.

It is noteworthy that CPCO, CPSO, and CPRO were the richest sources of TTC, containing approximately two times higher TTC (70.21–76.98 mg/100 g) than the CPLO (TTC = 44.04 mg/100 g) and CPMTO (TTC = 46.09 mg/100 g). However, TTC in CPPO was at the moderate level (64.45 mg/100 g). The results of TTC and individual tocopherols in the investigated oils demonstrated a wide variability. Evidence from large observational studies indicates that the climate during the growth and ripening of oilseeds, their genetic varieties, cold-pressing parameters, and oil storage conditions can affect amounts of tocopherols in CPOs [3,32,33].

Other researchers noticed similar TTC results in different CPOs. For instance, TTC values in CPLO, CPSO, CPRO, CPCO, CPPO, and CPMTO were 38.7–162.4 mg/100 g, 68.9–75.5 mg/100 g, 63.8–66.0 mg/100 g, 70.0–85.0 mg/100 g, 92.6 mg/100 g, and 42.9 mg/100 g, respectively [14,18,32,34].

Some recent studies have suggested that the antioxidant properties of tocopherol homologues were dependent on their concentrations in bulk oils. α-Tocopherol had maximum antioxidant activity at 100 mg/kg, whereas higher amounts of γ-tocopherol (250–500 mg/kg) revealed the best antioxidant activity in bulk oil without a prooxidant effect [4]. However, exceeding the optimum concentration of α-tocopherol in oil resulted in the generation of α-tocopherol peroxy, α-tocopherol oxy, α-tocopherolquinone oxy, and hydroxy radicals [4,35].

It is evident that the α- and γ-tocopherol homologues dominated in all of the studied CPOs (Table 2). Our results showed the highest amount of α-tocopherol in CPSO (73.37 mg/100 g), whereas CPCO (1.20 mg/100 g) and CPLO (1.78 mg/100 g) contained the lowest concentration of α-tocopherol. The α-tocopherol content in CPMTO and CPRO was almost two-fold lower compared to CPSO (Table 2). This homologue has the highest nutritional value because of the specificity of absorption and distribution within the human body [3,33]. Interestingly, the selective accumulation of α-tocopherol is mediated by α-tocopherol-transfer protein (α-TTP) (hepatic cytosolic protein), which preferentially binds to α-tocopherol over other homologues. For this reason, α-tocopherol is metabolized relatively slowly than other forms, and it is present in more quantities in human plasma. Moreover, α-tocopherol accumulated in areas with maximum free radical production, especially in the mitochondrial membranes and endoplasmic reticulum in the lungs and heart [36].

Nutrition standards for the Polish population recommend an all-day intake of α-tocopherol of 8 and 10 mg for women and men, respectively. However, some countries recommend higher amounts of α-tocopherol, ranging between 11 and 15 mg per adult [37]. The daily recommendation of E vitamin can be met by eating 7 spoons (55.5 g) of CPRO, almost 5 spoons (38.6 g) of CPMTO, and 2.5 spoons (20.4 g) of CPSO. From this point of view, some of the tested CPOs appear to be rich sources of α-tocopherol, which can protect against atherosclerosis, cardiovascular diseases, cataracts, neural tube defects, and cancer [33].

It is noteworthy that the γ-tocopherol predominated in CPCO (74.27 mg/100 g), whereas CPLO, CPPO, and CPRO contained an approximately two times lower amount of this homologue (42.11–56.96 mg/100 g). Among all of the studied oils, the γ-tocopherol was only undetected in CPSO (Table 2).

In contrast to α-tocopherol, γ-tocopherol can more effectively prevent lipid peroxidation and limit the prooxidant effect of α-tocopherol. The γ-tocopherol is consumed more slowly than α-tocopherol due to its higher stability [34]. Furthermore, γ-tocopherol has unique anti-inflammatory activity relevant to chronic disease prevention compared to α-tocopherol [3,33].

Based on the obtained results, close connections can be observed between profiles of tocopherols and fatty acids of the investigated oils. The high amount of α-tocopherol is linked with C18:2, while γ-tocopherol content is associated with the presence of C18:3. This can be explained by the fact that oil containing high PUFA content is highly prone to oxidation, and tocopherols can inhibit these undesirable reactions [3,4]. The obtained results confirmed that CPLO and CPCO contained the highest concentrations of C18:3 (52.12 and 30.29%) and γ–tocopherol (42.26 and 74.27 mg/100 g) (Table 1 and Table 2). Moreover, CPSO and CPMTO were characterised by high amounts of C18:2 (57.64 and 56.46%) and α-tocopherol (73.37 and 38.91 mg/100 g). Unexpectedly, relationships between concentrations of fatty acids and tocopherol forms in CPPO were not observed. The C18:2 (50.39%) and γ-tocopherol (56.96 mg/100 g) were predominated in the studied CPPO. Other researchers noticed similar results in terms of the C18:2 (36.2–62.8%) and γ-tocopherol (52.3–644 µg/g) contents in pumpkin seed oils [38,39].

According to this study, β-tocopherol was detected only in CPMTO and CPSO in concentrations of 2.84 and 2.56 mg/100 g, respectively (Table 2). Moreover, β- and γ-tocopherols have similar reactivity with peroxyl radicals, while β-tocopherol does not inhibit smooth muscle proliferation [33,40]. 

Among all tocopherol homologues, δ-tocopherol was detected only in CPRO (1.10 mg/100 g) and CPCO (1.51 mg/100 g) (Table 2).

A recent studies have demonstrated that δ-tocopherol characterised more potent antiradical properties than γ-tocopherol [4]. For comparison, Gliszczyńska-Świgło et al. [33] reported that the concentration of δ-tocopherol in edible plant oils (except soybean oil) did not exceed 30 mg/kg.

The results of tocopherol profiles obtained in this study generally agree with the previously published data by other authors. Similar amounts of α-tocopherol (26.2–27.2 and 63.5–72.2 mg/100 g), γ-tocopherol (35.9–38.9 and 0.7–3.1 mg/100 g), β-tocopherol (not detected and 2.3–2.7 mg/100 g), and δ-tocopherol (0.8–0.9 mg/100 g and not detected) in CPRO and CPSO respectively were found by Franke et al. [32]. However, 17 different oils cold-pressed from rapeseed contained α-, γ-, and δ-tocopherols varying from 114.3 to 324.7 mg/kg, 155.1–508.2 mg/kg, and 5.3–18.0 mg/kg, respectively [16]. As expected, five extra virgin olive oil (EVOO) samples tested by the same authors had lower mean amounts of α- and γ-tocopherols (181.2 and 13.8 mg/kg), while δ-tocopherol was undetected. Moreover, α-, γ-, and δ-tocopherols in CPLO after 150 days of storage ranged between 0.8–3.2 mg/100 g, 28.1–41.0 mg/100 g, and 0.3–0.8 mg/100 g, respectively [18]. Generally, the tocopherol profile (α-tocopherol = 204.1 mg/kg, γ-tocopherol = 55.5 mg/kg, δ-tocopherol = 14.6 mg/kg) of CPMTO reported by Grajzer et al. [4] was similar, while levels of these homologues (α-tocopherol not detected, γ-tocopherol = 817.7 mg/kg, δ-tocopherol = 126 mg/kg) in CPCO were slightly higher than results presented in this study.

#### 2.1.3. Sterol Profiles

The sterol compositions and the total sterol contents (TSC) in the six investigated oils are presented in Table 3. 

Differences in TSC and the individual sterol compositions were evident for different CPO samples. The highest concentration of total sterols (684 mg/100 g) was revealed for CPRO, while CPPO had the lowest TSC (300 mg/100 g). Thus, CPRO was within the permissible TSC limits (450–1130 mg/100 g) quoted under the Codex Alimentarius [1]. However, Grajzer et al. [4] found approximately two times higher overall median quantity of total sterols (5459.9 mg/kg) in six commercially available CPPO samples.

From a nutritional and healthy point of view, sterols delay or inhibit the lipid oxidation process by scavenging free radicals. Moreover, they can reduce the LDL cholesterol fraction in human blood [41]. There is no doubt that the intake of sterols decreases LDL cholesterol and protects against cardiovascular diseases. On the other hand, sterol profile analyses can be used as a detection tool for adulteration of CPOs [42].

The sterol profiles of the tested CPOs can be affected by several factors, such as various cultivars, and growing, processing and storage conditions.

It can be noted that the β-sitosterol was the predominant compound in studied oils, with the highest amount in CPRO (336 mg/100 g), whereas CPLO (166 mg/100 g) comprised the lowest content of this sterol (Table 3). Moreover, a CPCO was characterised by a high level of β-sitosterol (265 mg/100 g). Szterk et al. [43] found a somewhat higher content of β-sitosterol in crude *Camelina sativa* oil (361.3 mg/100 g) and crude linseed oils (162.5 mg/100 g). However, refined rapeseed oil contained insignificantly lower β-sitosterol concentration (324.7 mg/100 g) [43]. Unexpectedly, technological processes did not significantly influence the highest level of β-sitosterol (approximately 94%) in EVOO samples obtained using super pressure (SP), continuous extraction techniques (2P and 3P), and traditional extraction, respectively [44].

On the other hand, brassicasterol was only found in oils cold-pressed from oilseeds belonging to the family of *Brassicaceae*, including CPRO (73 mg/100 g) and CPCO (22 mg/100 g), while campesterol (80–249 mg/100 g) dominated in both mentioned oil samples and CPLO (Table 3). Krygier et al. [45] reported similar amounts of brassicasterol (69.4–89.3 mg/100 g) and campesterol (216.1–231.3 mg/100 g) in three commercial CPROs. Additionally, campesterol concentrations in CPLO (80 mg/100 g) and CPCO (110 mg/100 g) were in close agreement with those for 30 commercial cold-pressed flaxseed oils from various manufacturers available on the Polish market (64.94–115.36 mg/100 g), crude linseed (97.5 mg/100 g), and *Camelina sativa* (109.6 mg/100 g) oils analysed by Szterk et al. [43] and Mikołajczak et al. [46].

However, ∆-7-stigmasterol was the principal sterol in CPMTO and CPSO, comprising up to 30% of the TSC (Table 3). The sterols identified in lower concentrations were ∆-5-avenasterol (44 and 35 mg/100 g in CPLO and CPCO, respectively) and ∆-7-avenasterol (57 and 21 mg/100 g in CPPO and CPMTO, respectively).

#### 2.1.4. Total Phenolic Content

Phenolic compounds have strong antioxidant properties due to their ability to reduce other compounds, singlet oxygen quenching, hydrogen donating and metal chelating. On the other hand, polyphenols can behave as anti-inflammatory, anticancer and antibacterial agents. Therefore, phenolic compounds inhibit the oxidation reactions in oils, as well as affect their sensory and nutritional properties. It is known that CPOs are rich sources of phenolic compounds because they are not removed during a refining process [4,20,27]. 

The results of TPC in six CPOs analysed by the Folin–Ciocalteu (F–C) method are summarized in Table 4.

This analytical method is prevalent because it allows determining all phenolic compounds, regardless of their structure. The TPC values in the tested oils varied from 2.93 mg GA/100 g for CPLO to 8.32 mg GA/100 g for CPPO. The obtained results demonstrated that the type of raw material affected total phenolic concentrations in the studied CPOs. Insignificant differences in TPC results (4.93–5.42 mg GA/100 g) were observed between CPMTO, CPSO, and CPRO, while CPCO contained significantly lower amounts of phenolic compounds (TPC = 4.17 mg GA/100 g) (Table 4, Duncan test).

Interestingly, other researchers [4,20,27] noticed higher amounts of total polyphenols in CPLO (37.57–84.9 mg caffeic acid (CA)/kg, 60.25–115.12 mg/100 g, 60.3–89.6 mg ferulic acid (FA)/100 g), CPPO (53.67–184.6 mg CA/kg, 41.7–55.6 mg FA/100 g), CPMTO (71.7–124.7 mg CA/kg, 45.4–50.7 mg FA/kg), CPCO (34.12–138.9 mg CA/kg, 90.2–120.1 mg FA/100 g), CPRO (50.7–112.8 mg FA/100 g), and CPSO (43.8–52.3 mg FA/100 g) from Polish manufacturers or produced and purchased during their shelf life on the Polish market. However, the mean TPC (2.1 mmol GA/kg) in 17 samples of CPRO from England, Ireland, France, and other European Union countries was lower than the mean TPC (3.9 mmol GA/kg) in 5 samples of EVOO from Italy, Greece, and Spain [16].

#### 2.1.5. Antioxidant Activity

The AA results obtained by 2,2-diphenyl-1-picrylhydrazyl (DPPH) and 2,2’-azino-bis(3-ethylbenzothiazoline-6-sulfonic acid) (ABTS) methods demonstrated differences in the antioxidant abilities of the studied CPOs to scavenge/deactivate DPPH and ABTS radicals. Moreover, the ferric reducing antioxidant power (FRAP) spectrophotometric single electron transfer (SET)-based assay measured the capacity of oil antioxidants to reduce an oxidant, which changed colour when reduced.

It is noteworthy that the AA results determined using three analytical methods, such as DPPH, ABTS, and FRAP, that differed significantly (by more than two orders of magnitude) for the same oil (Table 4). The differences between AA values for the studied CPOs can be explained by different mechanisms of the chosen analytical methods and their different affinities toward hydrophobic and hydrophilic antioxidants. The ABTS test was suitable for analysing both hydrophilic and lipophilic antioxidants, while DPPH measured antioxidants insoluble in water. In contrast, FRAP allows the quantification of the most hydrophilic antioxidants with redox potential not lower than that of the redox pair Fe^3+^/Fe^2+^ [47]. For this reason, the FRAP values (61.93–119.21 µmol TE/100 g) of the studied CPOs were approximately 2–5 and 13–18 times lower than those of DPPH (185.36–396.63 µmol TE/100 g) and ABTS (958.59–1638.58 µmol TE/100 g), respectively (Table 4). Moreover, the ABTS cation radical scavenging ability of tested CPOs was 4 to 6 times higher than their DPPH radical scavenging activity. These differences indicated that CPOs contained many bioactive compounds that can be classified as lipophilic antioxidants (e.g., tocopherols). The same tendency was observed by Symoniuk et al. [23], although lower differences (1.5–2.8 times) between ABTS and DPPH results for CPLO, CPPO, CPRO, CPCO, and CPMTO were found.

As can be seen in Table 4, CPPO had the highest AA values determined using all analytical methods (DPPH = 396.63 µmol TE/100 g, ABTS = 1638.58 µmol TE/100 g, and FRAP = 119.21 µmol TE/100 g). However, CPLO revealed the lowest DPPH (185.36, µmol TE/100 g), while ABTS (958.59 µmol TE/100 g) and FRAP (61.93 µmol TE/100 g) were the lowest for CPMTO. Insignificant differences were observed between the FRAP results of CPMTO and CPSO, as well as CPLO and CPCO. In addition, the Duncan test indicated that ABTS values of CPLO and CPSO as well as CPRO and CPCO were similar (Table 4). Moreover, DPPH results of CPMTO and CPSO as well as CPPO and CPCO did not differ significantly. The results obtained by the DPPH method for CPLO (185.36 µmol TE/100 g), CPMTO (234.65 µmol TE/100 g) and CPSO (241.06 µmol TE/100 g) were in agreement with those (DPPH = 1.97–2.01, 2.14–2.56, and 1.76–2.34 mM TE/kg for CPLO, CPMTO, and CPSO, respectively) reported by Symoniuk et al. [20]. On the contrary, the same researchers reported lower DPPH values for CPPO (1.95–1.96 mM TE/kg), CPRO (1.81–1.96 mM TE/kg), and CPCO (1.88–2.44 mM TE/kg) than those demonstrated in our work. Unexpectedly, mean ABTS values for 17 oils cold-pressed from rapeseed (48.8 mmol TE/kg) and five samples of EVOO (50.9 mmol TE/kg) were lower than mean DPPH results (74.2 and 58.1 mmol TE/kg for CPROs and EVOOs, respectively) [16].

These AA differences can be explained by the fact that the antioxidant properties of CPOs were affected by many factors, such as agronomic, genetic, and environmental conditions, technological processing parameters, as well as differences in the mechanism of the applied assays for measuring their total antioxidant potential.

### 2.2. Oxidative Stability and Quality of Cold-Pressed Oils

#### 2.2.1. Oxidative Stability 

Oxidative stability is one of the most important parameters describing the susceptibility of oil to oxidation, and it is associated with its shelf life. 

The oxidative stability of the studied CPOs was determined using the Rancimat method and expressed as the induction period (IP) at 100 °C (Table 5).

It can be noted that the IP values varied from 4.87 h for CPLO to 12.93 h for CPRO. This suggests that CPRO had the highest resistance to oxidation reactions among the investigated oils. Other researchers [20,27] also reported similar IP results for CPRO (12.96–13.98 h) and CPLO (2.85–4.96 h). Evidently, the obtained IP results demonstrated that the oxidative stability of the studied oils was correlated with their degree of unsaturation. The high oxidative stability of CPRO was most likely due to its FAC with the lowest PUFA content (27.94%). In contrast, the shortest IP (4.87 h) was achieved in the case of CPLO containing the highest PUFA level (68.42%) and the lowest total amounts of tocopherols (44.04 mg/100 g) and phenolics (2.93 mg GA/100 g). Moreover, a high percentage of C18:3 (30.29%) caused the CPCO to oxidize quickly, exhibiting a low IP value (5.37 h). The oxidative stability of CPCO was similar to IP results (4.8–6.8 h) of different CPCOs reported in our previous work [8] and those (4.26–6.18 h) reported by Ratusz et al. [28].

Insignificant differences (*p* > 0.05, Duncan test) in IP values (9.47, 9.03, and 9.23 h) were observed between CPPO, CPMTO, and CPSO containing similar content of PUFA (50.59, 56.76, and 57.74%, respectively).

For comparison, CPPO, CPMTO, and CPSO analysed by other authors [20,23,43,48] had different IP results (13.63–34.39, 5.47–11.17, and 6.13–19.87 h, respectively). These differences can be explained by the fact that the IP values are highly dependent on the Rancimat test parameters (temperature and airflow rate) and profiles of fatty acids, antioxidants, oxidation products, and impurities in oil samples.

Additionally, the IP results of the investigated CPOs determined using the Rancimat method increased in the opposite order of their calculated COX values (Table 1 and Table 5).

#### 2.2.2. Amounts of Primary and Secondary Oxidation Products and Free Fatty Acids

Oxidative parameters such as peroxide value (PV), p-anisidine value (pAnV), total oxidation value (TOTOX), acid value (AV), and free fatty acids (FFA) were used to determine the oxidation state of the analysed CPOs. 

Primary oxidation processes in oils mainly form hydroperoxides, (measured via PV), while secondary oxidation occurs when the peroxides decompose to form aldehydes and ketones (measured via pAnV). The TOTOX calculated from the PV and pAnV data is a measure of the overall oxidation profile of each oil. However, AV determines the amount of FFA, released from the triacylglycerol molecules during processing and storage conditions. These important oxidative indicators affecting the CPO quality are presented in Table 5.

As can be seen, amounts of the primary oxidation products in all of the studied CPOs (PV = 0.24–4.61 meq O_2_/kg) were below the legal limit (15 meq O_2_/kg) permitted for cold-pressed and virgin oils, according to Codex Alimentarius [1]. Interestingly, CPLO, CPRO, and CPCO reached one-order lower PV results (0.24–0.61 meq O_2_/kg) when compared to other studied oils (PV = 2.44–4.61 meq O_2_/kg) (Table 5). In addition, PV for CPSO (4.61 meq O_2_/kg) was approximately two times higher than those obtained for CPPO and CPMTO (PV = 2.44 and 2.88 meq O_2_/kg, respectively).

The PV results below 1.0 meq O_2_/kg for CPRO (PV = 0.85 meq O_2_/kg) and CPLO (PV = 0.95 meq O_2_/kg) were also observed by Symoniuk et al. [23]. On the contrary, the content of primary oxidation products (PV = 4.91 meq O_2_/kg) in camelina oil analysed by the same authors was higher than PV (0.24 meq O_2_/kg) for the CPCO sample tested in this work. However, PV (0.42 meq O_2_/kg) for CPRO was significantly lower than those reported by McDowell et al. [16] for 17 various oils cold-pressed from rapeseed (mean PV = 5.5 meq O_2_/kg) and five samples of EVOO (mean PV = 10.4 meq O_2_/kg). Moreover, PV results for EVOOs produced by different technologies, namely, super pressure (SP), two-phase (2P), and three-phase (3P) systems, and traditional extraction system ranged between 16.10–19.40 meq O_2_/kg [44]. This can be explained by the fact that lipid oxidation is a complex process, depending on many factors, such as oilseed variety and quality, the FAC of the pressed oil and the processing technology applied. In addition, the content of peroxides in oils can depend on their shelf life. The PV generally increases with the storage time [16]. Therefore, oils with low PV values prolong their shelf life before consumption [49]. The PV of oil is also more closely associated with its IP determined using the Rancimat test; thus, oil with initially low PV is more prone to oxidation processes than oil containing high levels of peroxides [23,50].

It is interesting to note that amounts of secondary oxidation products in CPRO, CPCO, and CPPO were higher (pAnV = 0.88–4.77) than contents of primary oxidation products in these oils (PV = 0.24–2.44 meq O_2_/kg) (Table 5). This suggests that the primary oxidation products were decomposed to secondary oxidation products in the mentioned oils. Unexpectedly, CPLO contained the lowest amounts of secondary oxidation products (pAnV = 0.39), while pAnV (4.77) was the highest for CPPO. For comparison, other authors [23] reported significantly higher pAnV (7.14) for CPPO. Moreover, CPOs analysed by various researchers had pAnV ranging between 0.07–1.43, 0.58–8.60, 0.13–2.56, 0.53–3.55, 0.22–1.93, and 0.45–0.89 for CPLO, CPPO, CPMTO, CPRO, CPCO, and CPSO, respectively [4,5,20,23,25,27].

The calculated TOTOX indexes showed different degrees of oxidation, varying from 1.61 for CPLO to 10.01 for CPSO (Table 5). Based on obtained TOTOX results, all of the studied oils revealed acceptable quality, although the overall oxidation profiles of CPSO (TOTOX = 10.01) and CPPO (TOTOX = 9.65) were significantly higher than the oxidative status of other oils (TOTOX < 6.5).

Similar TOTOX results (2.38–11.74) for oils cold-pressed from linseed, milk thistle, rapeseed, camelina, and sunflower seeds were reported by other authors [20,23,27].

Furthermore, AV and FFA levels in the tested CPOs can be good indicators of their status in terms of oxidative stability. A higher concentration of triacylglycerol hydrolysis products indicates a lower oil quality level. The AV (0.31 and 0.37 mg KOH/g) and FFA (0.15 and 0.18%) results were the lowest for CPCO and CPLO and did not differ significantly among each other (Duncan test, Table 5). On the contrary, CPMTO had the highest results of AV (2.83 mg KOH/g) and FFA (1.42%). Nevertheless, the amounts of hydrolysis products in CPRO (AV = 0.42 mg KOH/g and FFA = 0.23%) were approximately three times lower than those in CPSO (AV = 1.16 mg KOH/g and FFA = 0.58%) and CPPO (AV = 1.55 mg KOH/g and FFA = 0.77%).

Krygier et al. [45] and Symoniuk et al. [20] reported similar AV values for CPRO (0.29–2.46 mg KOH/g) and CPSO (0.40–1.40 mg KOH/g), whereas Meddeb et al. [51] observed much higher AV results (5.48–8.34 mg KOH/g) for oils cold-pressed from some varieties of milk thistle seeds growing in different areas in Tunisia. However, CPRO samples from various geographic locations, such as England, Ireland, France, and other European Union countries, had comparable mean AV value (2.2 mg KOH/g) with that of EVOOs (mean AV = 2.0 mg KOH/g) [16]. Moreover, the type of technological process affected the acidity of EVOOs (0.54–0.72%) [44]. These results were in agreement with FFA levels analysed in CPSO (0.58%) and CPPO (0.77%).

Importantly, all of the tested oils had AV values below the maximum permissible limit (4.0 mg KOH/g) prescribed by the Codex Alimentarius Commission [1].

It is well known that oil acidity is related to the presence of FFAs, initially formed during enzymatic hydrolysis caused by lipases, naturally occurring in seeds. For this reason, AV depends on the general quality, varieties, and humidity of seeds, the shelf life of oils pressed from them, and their light exposure during storage in bottles [8,52,53]. The FFAs are much more susceptible to oxidation than those connected to a triacylglyceride moiety, causing oil deterioration and influencing sensory characteristics; thus, high AV values are not desired for total oil quality [51].

The consumption of lipid oxidation products may cause serious health risks to consumers due to their reactions with proteins, DNA, and phospholipids, which promote chronic diseases such as inflammation, cancer, and cardiovascular diseases [54].

#### 2.2.3. Water and Volatile Matter Contents

It can be noted that levels of water and volatile compounds (WVC) in the investigated CPOs ranged between 0.020% and 0.090% (Table 5), which are below the legal requirement recommended by Codex Alimentarius (0.20%) [1]. 

Similar WVC content (779 ppm) in oil pressed from milk thistle seeds was observed by Rokosik et al. [30]. Somewhat higher WVC values (0.05–0.17%) were determined for eight CPCO samples produced by the most famous Polish manufacturers, as previously described in our report [8]. In addition, traditionally extracted EVOO revealed significantly lower humidity (0.04%) than super-pressure extracted EVOOs (0.07–0.15%) [44]. It is known that water excess in vegetable oils favours hydrolysis of triacylglycerols, causing oil deterioration. A high amount of water in oil can be observed when raw material is too wet, filtration after cold-pressing is inefficient, or the oil shelf life is prolonged.

#### 2.2.4. Polycyclic Aromatic Hydrocarbon Content

The concentrations of four polycyclic aromatic hydrocarbons (PAHs) (benzo(a)pyrene (B(a)P), chrysene (Chr), benzo(a)anthracene (B(a)A), and benzo(b)fluoranthene (B(b)F) in six CPOs were determined, and the obtained results are listed in Table 5. It should be emphasized that these compounds may be toxic to healthy tissues causing carcinogenic and mutagenic effects. Therefore, limits of 10 μg/kg for the sum of four PAHs (Σ4PAHs) and 2 μg/kg for B(a)P in vegetable oils were established by European Commission [55]. It is noteworthy that B(a)P (0.20–0.82 µg/kg) and Σ4PAHs (1.16–8.76 µg/kg) contents in the analysed oil samples were significantly lower than that recommended by the European Commission [55]. The CPLO revealed the highest amounts of Σ4PAHs (8.76 µg/kg) and B(a)A (7.16 µg/kg), while CPCO had the lowest levels of Σ4PAHs (1.16 µg/kg) and B(a)A 0.51 µg/kg). In addition, Chry concentration was high in CPLO (0.76 µg/kg) and CPMTO (0.83 µg/kg). However, the same Chry content (0.54 µg/kg) was observed for CPPO and CPSO (Table 5).

For comparison, amounts of Σ4PAHs in rapeseed oils pressed from conventional and organic farming seeds varied from 3.13 to 6.15 µg/kg [56]. Significantly higher concentrations of total PAHs (17.85–37.16 μg/kg) in different cold-pressed vegetable oils were reported by Roszko et al. [57]. However, contamination of these oils with B[a]P (0.02–1.25 μg/kg) was similar to those found in our study.

All of the analysed CPOs had good quality due to low levels of dangerous compounds, such as PAHs affecting human health.

### 2.3. Relationships between Descriptive Attributes and Acceptance Test

**Figure 1:**
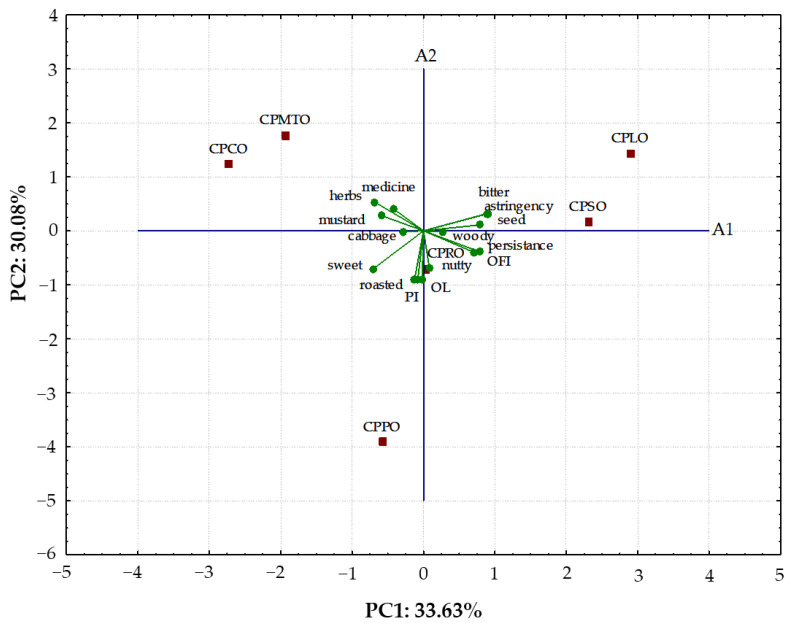
Principal component analysis of the sensory descriptors, hedonic overall liking (OL), purchase intent (PI), and overall flavour intensity (OFI) for six cold-pressed oils. Abbreviations: CPLO—cold-pressed linseed oil; CPPO—cold-pressed pumpkin oil; CPMTO—cold-pressed milk thistle oil; CPRO—cold-pressed rapeseed oil; CPCO—cold-pressed camelina oil; CPSO—cold-pressed sunflower oil.

The PCA was applied to find the relationships between descriptive attributes of the investigated CPOs, overall flavour intensity (OFI), and hedonic responses: overall liking (OL) and purchase intent (PI). The first two principal components accounted for 63.71% of the experimental data variance (Figure 1).

As can be seen in Figure 1, the CPPO characterised by very high scores of sweet taste and roasted and nutty flavour intensity created an evidently distinct cluster. Furthermore, the CPSO and CPLO having the highest bitterness perception were located close to each other on the right side of the map. Both CPMTO and CPCO were situated on the left side of the PCA map due to the presence of herb-like flavour in the sensory profile. Additionally, the medicine-like flavour was identified only in CPMTO, while differences in the mustard-like flavour were found for CPMTO and CPCO. However, CPRO with a dominance of cabbage-like and woody-like sensory attributes was located in the middle of the PCA map.

The current study found a high positive correlation (r = 0.9831, *p* < 0.001) between OL and PI. The attributes of sweet taste (r = 0.6387) and roasted flavour (r = 0.7187) contributed insignificantly (*p* > 0.05) positively to consumer acceptance (OL). These attributes appeared in CPPO and received the highest consumer scores. On the contrary, the attributes of bitter taste, astringency, and herbs-like flavour were responsible for the lower OL (r = −0.3601, −0.3404, and −0.4222, respectively, *p* > 0.05) and PI (r = −0.4470, −0.4307, and −0.4425, respectively, *p* > 0.05) of the tested CPOs.

### 2.4. Chemometrics Analysis

Multivariate mathematical approaches, such as PCA and HCA, were powerful tools that permitted a relatively simple representation of similarities and discrepancies between the investigated oil samples based on complex analytical and sensory data.

#### 2.4.1. Principal Component Analysis

**Figure 2:**
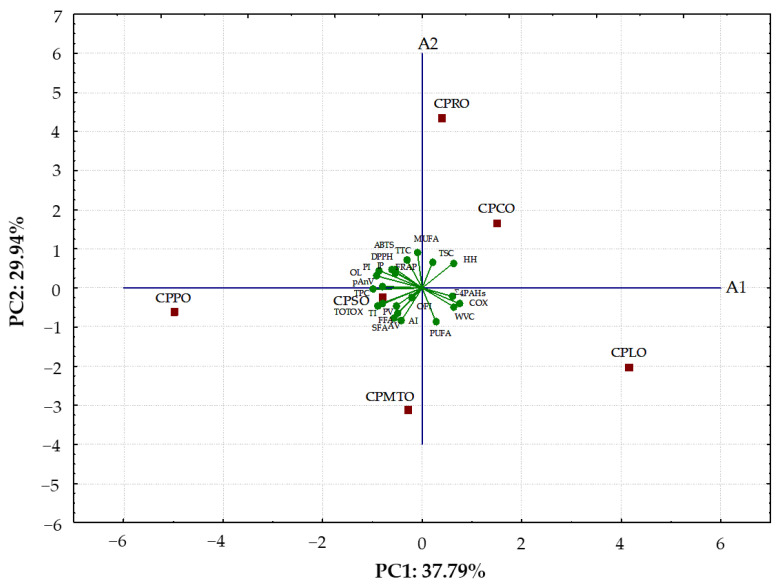
Biplot of scores and loadings of data obtained for physicochemical and overall sensory characteristics of six cold-pressed oils. Abbreviations: SFA—saturated fatty acids; MUFA—monounsaturated fatty acids; PUFA—polyunsaturated fatty acids; COX—calculated oxidisability value; AI—atherogenicity index; TI—thrombogenicity index; HH—ratio of hypocholesterolemic to hypercholesterolemic fatty acids; TTC—total tocopherol content; TSC—total sterol content; TPC—total phenolic content; DPPH—2,2-diphenyl-1-picrylhydrazyl method; ABTS—2,2′-azino-bis(3-ethylbenzothiazoline-6-sulfonic acid); FRAP—ferric reducing antioxidant power; IP—induction period; PV—peroxide value; pAnV—anisidine value; TOTOX—total oxidation value; AV—acid value; FFA—free fatty acids; WVC—water and volatile matter content; Σ4PAHs—sum of four specific polycyclic aromatic hydrocarbons; OL—overall liking; PI—purchase intent; OFI—overall flavour intensity; CPLO—cold-pressed linseed oil; CPPO—cold-pressed pumpkin oil; CPMTO—cold-pressed milk thistle oil; CPRO—cold-pressed rapeseed oil; CPCO—cold-pressed camelina oil; CPSO—cold-pressed sunflower oil.

PCA was applied to the matrix formed by all physicochemical parameters and three sensory characteristics corresponding to the studied CPOs. As can be seen in Figure 2, a biplot depicted the relationships between observed data (six CPOs: CPLO, CPPO, CPMTO, CPRO, CPCO, and CPSO) and dependent variables (SFA, MUFA, PUFA, COX, AI, TI, HH, TTC, TSC, TPC, DPPH, ABTS, FRAP, IP, PV, pAnV, TOTOX, AV, FFA, WVC, Σ4PAHs, OFI, OL, and PI) in terms of principal components. The first two principal components with the highest eigenvalue contributions (9.07 and 7.18, respectively) for the observed variations were taken from the 24 principal components. A combination of PC1 and PC2 described 67.73% (PC1 = 37.79%, PC2 = 29.94%) of the total variance in the dataset. It was found that PC1 highly negatively correlated with TPC (−0.9918), OL (−0.9049), TI (−0.8813), and PI (−0.8591), while PC2 showed the highest positive correlation with MUFA (0.9175) and inversely contributed by PUFA (−0.8679) and AI (−0.8283).

The biplot depicted that CPRO and CPCO with the highest MUFA contents (64.83 and 35.33%) and the lowest amounts of SFA (7.12 and 9.72%), AI (0.02 and 0.03), and PV (0.42 and 0.24 meq O_2_/kg) were clustered in the right upper quarter. CPLO containing the highest levels of PUFA (68.42%), COX (13.14), Σ4PAHs (8.76 μg/kg), and OFI (6.83) and the lowest values of TTC (44.04 mg/100 g), TPC (2.93 mg GA/100 g), DPPH (185.36 μmol TE/100 g), IP (4.87 h), pAnV (0.39), TOTOX (1.61), OL (4.18), and PI (3.73) was also situated on the same side under the A1 axis. Interestingly, the lowest oxidative stability and antioxidant contents in CPLO as well as the highest OFI affected low scores of its acceptance (via low scores of OL and PI). However, CPPO, CPSO, and CPMTO containing similar high concentrations of SFA (11.20–19.53%), PUFA (50.59–57.74%), TI (0.23–0.45), TPC (5.25–8.32 mg GA/100 g), IP (9.03–9.47 h), PV (2.44–4.61 meq O_2_/kg), AV (1.16–2.83 mg KOH/g), FFA (0.58–1.42%), and Σ4PAHs (2.12–2.58 μg/kg) were located in the bottom left quarter of the biplot (Figure 2). The PCA results revealed that the investigated oil samples were clearly categorised into three distinct groups according to different raw materials and their botanical origin, which indicated differences in determined physicochemical parameters and sensory characteristics.

#### 2.4.2. Hierarchical Cluster Analysis

**Figure 3:**
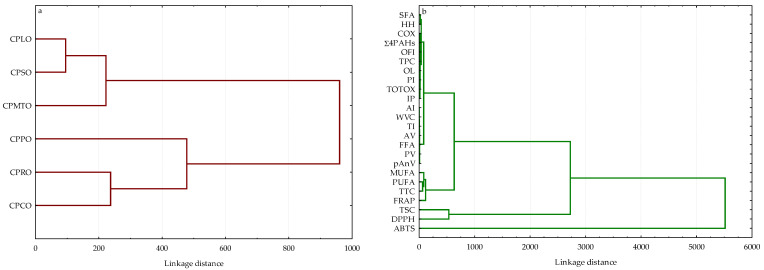
Dendrograms of hierarchical cluster analysis for (**a**) six cold-pressed oils and (**b**) the studied variables. Abbreviations: SFA—saturated fatty acids; MUFA—monounsaturated fatty acids; PUFA—polyunsaturated fatty acids; COX—calculated oxidisability value; AI—atherogenicity index; TI—thrombogenicity index; HH—ratio of hypocholesterolemic to hypercholesterolemic fatty acids; TTC—total tocopherol content; TSC—total sterol content; TPC—total phenolic content; DPPH—2,2-diphenyl-1-picrylhydrazyl method; ABTS—2,2′-azino-bis(3-ethylbenzothiazoline-6-sulfonic acid); FRAP—ferric reducing antioxidant power; IP—induction period; PV—peroxide value; pAnV—anisidine value; TOTOX—total oxidation value; AV—acid value; FFA—free fatty acids; WVC—water and volatile matter content; Σ4PAHs—sum of four specific polycyclic aromatic hydrocarbons; OL—overall liking; PI—purchase intent; OFI—overall flavour intensity; CPLO—cold-pressed linseed oil; CPPO—cold-pressed pumpkin oil; CPMTO—cold-pressed milk thistle oil; CPRO—cold-pressed rapeseed oil; CPCO—cold-pressed camelina oil; CPSO—cold-pressed sunflower oil.

The HCA was used to group the studied oils per their similarities and discrepancies based on 24 physicochemical and sensory variables. The dendrograms in Figure 3 showed the clustering patterns of six oils and variable sets. It can be noted that CPO samples were segregated into two main clusters (Figure 3a). The first cluster included an inter-cluster of CPLO, CPSO, and CPMTO. It is noteworthy that CPLO and CPSO had similar content of SFA (11.63 and 11.20%) and ABTS values (1040.86 and 1085.10 μmol TE/100 g), while CPMTO revealed somewhat lower ABTS (958.59 μmol TE/100 g). In addition, insignificant differences between TPC (5.42 and 5.25 mg GA/100 g), DPPH (234.65 and 241.06 μmol TE/100 g), FRAP (61.93 and 62.22 μmol TE/100 g), and IP (9.03 and 9.23 h) were observed for CPMTO and CPSO. Evidently, CPCO, CPRO, and CPPO were arranged in the second cluster. This group was associated with the highest AC (DPPH = 293.10–396.63 μmol TE/100 g, ABTS = 1328.00–1638.58 μmol TE/100 g, and FRAP = 75.80–119.21 μmol TE/100 g) together with high TTC (64.45–76.98 mg/100 g) in CPCO, CPRO, and CPPO. CPPO was distanced from this cluster because this oil sample had approximately two times higher amounts of TPC (8.32 mg GA/100 g) and SFA (18.92%) as well as sensory acceptance score (OL = 8.21) than those of CPRO and CPCO (TPC = 4.17–4.93 mg GA/100 g, SFA = 7.12–9.72%, OL = 5.00–6.72).

On the other hand, cluster analysis results indicate that the determined 24 physicochemical and sensory parameters as variables comprised three main groups (Figure 3b). The dendrogram clearly separated the ABTS method allowing the determination of both hydrophilic and lipophilic antioxidants present in oil samples from the other variables. However, DPPH method and TSC formed one cluster. This suggests that hydrophobic compounds such as sterols influenced the DPPH results of the investigated CPOs. However, the third cluster was composed of two subgroups consisting of (I) FRAP, TTC, PUFA, and MUFA and (II) all studied oxidative stability parameters (five characteristic quality values, oxidisability value, three nutritional quality indexes, IP), sensory characteristics (OFI, OL, PI), TPC, SFA, and amounts of undesirable compounds (Σ4PAHs and WVC). Unexpectedly, the results of HCA depicted that lipophilic antioxidants, such as tocopherols (TTC), PUFA, and MUFA levels, affected the FRAP values of the studied CPOs (Figure 3b). In addition, phenolic compounds (TPC) contributed more to sensory characteristics (OFI, OL, and PI) of CPOs than impurities (Σ4PAHs, WVC), oxidation and hydrolysis products (PV, pAnV, TOTOX, IP, COX, AV, FFA), nutritional quality indexes (AI, TI, HH), and SFA content. Considering the Euclidean distances, the HCA may capture physicochemical and sensory similarities between groups of the studied CPOs and variables more efficiently visually than the PCA.

#### 2.4.3. Correlation Analysis

**Figure 4:**
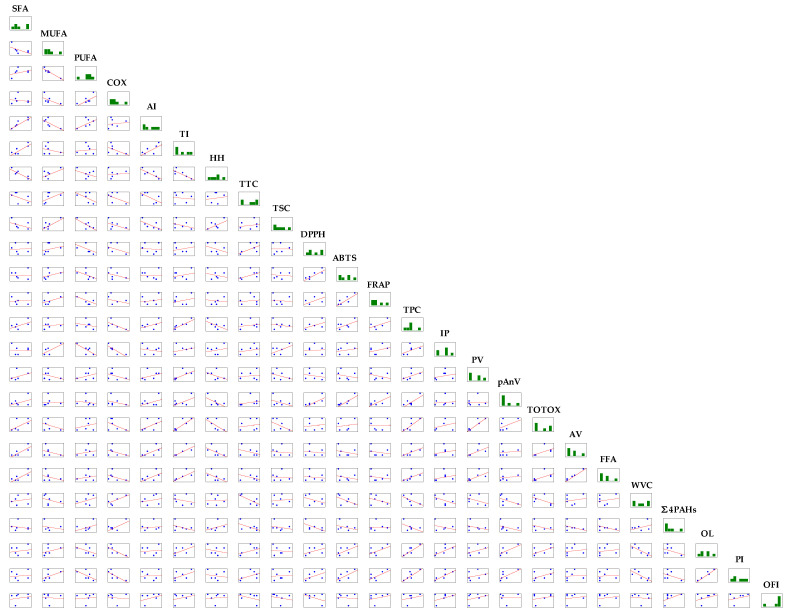
Correlation matrix between physicochemical parameters and sensory quality of six cold-pressed oils. Abbreviations: SFA—saturated fatty acids; MUFA—monounsaturated fatty acids; PUFA—polyunsaturated fatty acids; COX—calculated oxidisability value; AI—atherogenicity index; TI—thrombogenicity index; HH—ratio of hypocholesterolemic to hypercholesterolemic fatty acids; TTC—total tocopherol content; TSC—total sterol content; TPC—total phenolic content; DPPH—2,2-diphenyl-1-picrylhydrazyl method; ABTS—2,2′-azino-bis(3-ethylbenzothiazoline-6-sulfonic acid); FRAP—ferric reducing antioxidant power; IP—induction period; PV—peroxide value; pAnV—anisidine value; TOTOX—total oxidation value; AV—acid value; FFA—free fatty acids; WVC—water and volatile matter content; Σ4PAHs—sum of four specific polycyclic aromatic hydrocarbons; OL—overall liking; PI—purchase intent; OFI—overall flavour intensity.

The positive and negative correlations between physicochemical, nutritional, and sensory characteristics of six tested CPOs are presented as a correlation matrix in Figure 4.

It is noteworthy that positive significant relationships were found between SFA–AI (r = 0.9257, *p* = 0.008), SFA–TI (r = 0.8714, *p* = 0.024), SFA–AV (r = 0.8630, *p* = 0.027), and SFA–FFA (r = 0.8553, *p* = 0.030), whereas SFA negatively associated with HH ratio (r = −0.8815, *p* = 0.020). This suggests that nutritional quality indexes (AI and TI) and acidity indexes (AV and FFA) increased with increasing SFA content in CPOs. Thus, the intake of oils rich in SFA increases the risk of cardiovascular diseases.

Expectedly, COX values providing information about the oxidative stability of six CPOs inversely correlated with their IP results determined by the Rancimat method (r = −0.8810, *p* = 0.020). Moreover, a high negative correlation was found between MUFA and PUFA (r = −0.9676, *p* = 0.002), while TSC positively affected the MUFA amounts in the studied oils (r = 0.8200, *p* = 0.046). Additionally, the calculated r values indicated significant associations among AA results of all oils determined using ABTS and DPPH (r = 0.8899, *p* = 0.018) as well as ABTS and FRAP (0.8699, *p* = 0.024) methods. This can be explained by the fact that at the same time, antioxidants in the tested oils were capable of reducing ferric-tripyridyltriazine (Fe^3+^-TPTZ) to an intense blue colour ferrous-tripyridyltriazine complex (Fe^2+^-TPTZ) and scavenging ABTS and DPPH radicals. However, the effect of TPC on AA of six CPOs determined using three analytical methods was insignificant (r = 0.5629–06428, *p* > 0.05). This suggests that phenolic compounds in the studied oils contributed insignificantly to their AA. In contrast, high correlation coefficients were observed between TPC–OL (r = 0.8797, *p* = 0.021), TPC–PI (r = 0.8414, *p* = 0.036), and TPC–TI (r = 0.8986, *p* = 0.015). Unexpectedly, TPC significantly influenced the prothrombotic index and enhanced consumer acceptance, purchase intent, and positive emotions. However, these hedonic sensory scores significantly decreased with increasing water content in the tested CPOs (r = −0.8400, *p* = 0.036 and r = −0.8132, *p* = 0.049 for relationships OL—WVC, PI—WVC, respectively). It can be seen that the high sensory acceptability (OL) was essential for the consumer to intend to buy (PI) the CPOs (r = 0.9831, *p* < 0.0001).

Further studies are required to understand better interactions and relationships between bioactive compounds, other physicochemical, and sensory parameters of studied CPOs to predict their nutritional effectiveness and health benefits.

## 3. Materials and Methods

### 3.1. Reagents and Samples

All of the reagents and chemicals utilized in this research were purchased from Merck Sp. z o. o. (Warszawa, Poland). Six different CPOs in the original sealed dark glass bottles (250 mL) were donated by domestic manufacturers, which are leaders in the Polish market. These oil samples were obtained by mechanically pressing unheated seeds without additional extraction with solvents, followed by washing with water, precipitation, filtration, and centrifugation. The oils with a valid shelf life of at least 6 months were coded (CPLO—cold-pressed linseed oil, CPPO—cold-pressed pumpkin oil, CPMTO—cold-pressed milk thistle oil, CPRO—cold-pressed rapeseed oil, CPCO—cold-pressed camelina oil, and CPSO—cold-pressed sunflower oil) and kept refrigerated (4 °C) until analysis.

### 3.2. Determination of Fatty Acid Compositions

The FAC of each CPO was determined according to the official ISO 5508:1996 method [58]. Fatty acid methyl esters were prepared in accordance with the standard ISO 5509:2000 procedure [59] and analysed using a gas chromatography (HP 5890 GC) fitted with capillary column BPX 70 (60 m × 0.25 mm, 0.25 µm) and equipped with a flame-ionization detector (FID) (Hewlett-Packard, Avondale, PA, USA). The furnace temperature at 1.3 °C/min was 210 °C, initially starting from 150 °C. The temperatures of the injector and the detector were set at 250 °C. Helium was used as the carrier gas at a flow rate of 0.6 mL/min. The individual peaks were identified by comparison of their retention times with those of fatty acid methyl ester standards.

### 3.3. Calculated Oxidisability (COX) Value and Nutritional Quality Indexes

The *COX* values of the studied CPOs were calculated based on the Formula (1) proposed by Fatemi and Hammond [60].

(1) $$COX=\frac{C18:1+10.3\times C18:2+21.6\times C18:3}{100}$$

Nutritional quality indexes, including *AI* and *TI*, were calculated by Formulas (2) and (3), respectively, given by Ulbricht and Southgate [24].

(2) $$AI=\frac{C12:0+4\times C14:0+C16:0}{\Sigma MUFA+\Sigma \left(\omega -3\right)+\Sigma (\omega -6)}$$

(3) $$TI=\frac{C14:0+C16:0+C18:0}{0.5\times MUFA+0.5\times \Sigma \left(\omega -6\right)+3\times \Sigma \left(\omega -3\right)+(\frac{\omega -3}{\omega -6})}$$

However, the *HH* ratio was calculated according to the Formula (4) proposed by Santos-Silva et al. [61] to evaluate the impact of fatty acids on human cholesterol levels.

(4) $$HH=\frac{C18:1+C18:2+C18:3+C18:4+C20:4}{C14:0+C16:0}$$

### 3.4. Determination of Tocopherol Compositions

The tocopherol compositions of the studied CPOs were determined chromatographically according to the official ISO 9936:2016 method [62] with some modifications. The oil sample (0.5 g) was dissolved in hexane (95 mL), injected (5–20 µL) into LiChrospher 100 Diol (125 × 4 mm, 5 µm particle size) column (Merc, Darmstadt, Germany), and analysed using Agilent 1100 HPLC system (Agilent Technologies, Palo Alto, CA, USA) with an autosampler and fluorescence detector. The mobile phase was a mixture of hexane with tetrahydrofuran (96:4, *v*/*v*%) at a flow rate of 0.8 mL/min. The fluorescence detector was set at 280 nm excitation wavelength and 340 nm emission wavelength. The tocopherol homologues were identified by comparison of the retention times with standards of the α-, β-, γ-, and δ-tocopherols.

### 3.5. Determination of Sterol Compositions

The sterol compositions were analysed using a gas chromatography (GC) according to the official ISO 12228-1:2014 method [63]. Each studied oil sample was saponified by adding methanolic potassium hydroxide solution (c = 1 mol/L). The sterol fraction was extracted with a hexane/methyl tert-butyl ether (1:1) mixture. A capillary gas chromatography (Agilent Technologies 6890, Wilmington, DE, USA) system equipped with a capillary column (25 m, 0.20 mm i.d. and 0.33 µm film thickness) and a flame ionization detector (FID) was applied for separation and quantification of the silylated sterol fraction. The temperatures of the injector and detector were both set at 300 ℃. The carrier gas was hydrogen at a 1.5 mL/min flow rate. The sterols were identified by comparing the retention times with those of standard samples, and 5α-cholestane was used as an internal standard for quantification. 

### 3.6. Determination of Total Phenolic Content and Antioxidant Activity

The methanolic extracts of oils were prepared for TPC and AA analysis based on the previously reported procedure [15]. Briefly, 2.00 g of oils were weighed in test tubes and extracted with 5 mL of methanol for 30 min using an orbital shaker (SHKA25081 CE, Labo Plus, Warszawa, Poland). Then, the samples were stored in a refrigerator for 30 min to separate methanolic extracts from the oil layers. The extraction was repeated three times, whereas obtained extracts were combined and collected into glass bottles.

The TPC and AA of oil samples were determined using the F–C, DPPH, ABTS, and FRAP methods, respectively, according to procedures described in our previous work [64]. The UV–Vis spectra were recorded using a Hitachi U-2900 spectrophotometer (Hitachi, Tokyo, Japan) in a 1 cm quartz cell. The TPC results were expressed as mg of gallic acid equivalents per 100 g of oil sample (mg GA/100 g), while the AA was expressed as micromoles of Trolox equivalents per 100 g of oil sample (μmol TE/100 g).

### 3.7. Determination of Oxidative Stability

The oxidative stability of each studied CPO was analysed with 743 Rancimat apparatus (Metrohm, Herisau, Switzerland) according to the AOCS official method Cd12b-92 [65]. The oil sample (3.00 ± 0.01 g) was oxidised by air (flow 20 L/h) at 100 ± 0.3 °C in a measuring cell supplied with distilled water. The conductivity of the oxidation products dissolved in the water was measured, and the induction period (IP) (expressed in hours) was determined.

The PV was determined potentiometrically by the official ISO 27107:2010 method [66] and expressed as milliequivalents of active oxygen per kilogram of oil (meq O_2_/kg).

The pAnV was measured spectrophotometrically according to the official ISO 6885:2016 method [67].

The TOTOX as a characteristic value of the total oxidation of triacylglycerols was calculated by the expression: TOTOX = (2PV + pAnV).

The amounts of FFA produced during the hydrolysis processes of oils and the AV were measured according to the official ISO 660:2020 method [68].

### 3.8. Determination of Water and Volatile Matter Contents

The WVC content in the analysed CPOs was determined by drying 10 g of oil in an oven at 103 °C according to the official ISO 662:2016 procedure [69].

### 3.9. Determination of Polycyclic Aromatic Hydrocarbons

A high-performance liquid chromatography with a fluorescence detector (HPLC-FLD, Shimadzu, Kyoto, Japan) was applied to determine four PAHs, including B(a)P, Chry, B(a)A, and B(b)F, in the studied CPOs. Each oil was dissolved in cyclohexane and extracted to dimethyl formaldehyde. Data acquisition and calculations were conducted in OpenLAB CDS ChemStation Edition Software version 10.1 (Agilent Technologies, Waldbronn, Germany). The reversed-phase Zorbax Eclipse PAH column (particle size 3.5 µm, length 150 mm, diameter 4.6 mm, Agilent, Santa Clara, CA, USA) with a precolumn Eclipse XDB-C18 (3.5 µm, 4.6 × 150 mm, Agilent) at an oven temperature of 30 °C were utilized for PAHs analysis in six CPOs. Benzo(b)chrysene diluted in acetonitrile was used as an internal standard. Calibration curves were prepared by using the peak areas as a function of the PAH concentration standards in the range between 0.25 and 8.50 µg/kg.

### 3.10. Hedonic Consumer Test

The consumer home-use test was conducted with 100 untrained consumers of both sexes aged between 21 and 65 years. The studied CPOs in 250 mL bottles were tested by consumers for six days (1 bottle per day). Overall acceptability (overall liking, OL) was evaluated by using the 9-point hedonic scale anchored by: 1 = “dislike extremely” and 9 = “like extremely”. Additionally, purchase intent (PI) was measured by using the 9-point scale (1 = “I would definitely not buy” and 9 = “I would definitely buy”). At the end of the tasting, consumers answered questions about their social demographic characteristics and CPOs’ consumption frequency. Before the tasting session, the sensory sensitivity of each consumer was screened and trained.

### 3.11. Sensory Profiling

The sensory profiling of each CPO was determined by quantitative descriptive analysis (QDA). Ten well-trained assessors (seven women and three men) evaluated thirteen sensory attributes (overall flavour intensity (OFI), two basic tastes, eight flavours, and two mouths feel sensory terms) of six investigated CPOs (Table 6). Each attribute was assessed using a 10 cm unstructured intensity scale anchored at the ends by “no intensity” on the left and “high intensity” on the right.

All of the studied oils were evaluated in duplicate by a trained sensory panel in a sensory laboratory under conditions described by ISO 8589:2007 [70]. Oil samples were labelled with random three-digit codes and served in a transparent glass jars (250 mL) at room temperature (about 20 °C). During each evaluation, warm dark tea and fresh apple were provided to panellists to clear their palates and avoid the carry-over effect. Before the tasting session, the sensory sensitivity of each panellist was screened and trained on sensory attributes typical for CPOs.

### 3.12. Statistical Analysis

All chemical analyses were conducted in triplicate on the same day. The obtained results were presented as mean (c) ± standard deviation (SD). The experimental data were statistically evaluated using analysis of variance (ANOVA) test. A post hoc Duncan test was applied to calculate the significant differences among the mean values of physicochemical and sensory parameters of oils at a probability level of *p* < 0.05.

Two chemometric techniques, such as PCA and HCA, were utilized to identify the possible links between the physicochemical, nutritional, and sensory characteristics of the studied CPOs. The scores and loadings of the data analysed using PCA were displayed as biplots. However, the Ward technique and the squared Euclidean distance matrix were performed to define each cluster, resulting in hierarchical dendrograms from HCA.

The statistical analysis was carried out using Statistica 8.0 software (StatSoft Inc., Tulsa, OK, USA), while Fizz software (Biosystemes, Courtenon, France) was used to collect all sensory data.

## 4. Conclusions

The similarities and discrepancies between amounts of the health-beneficial compounds (tocopherols, sterols, and polyphenols), total antioxidant potential, oxidative stability, compounds hazardous to health, and sensory attributes of the most popular CPOs in the Polish market were observed. The studied CPLO and CPCO were rich sources of PUFA with high content of health-promoted linolenic acid belonging to the ω-3 acid family (PUFA = 68.42 and 49.67%, C18:3 = 52.12% and 30.29%, respectively). However, CPPO, CPMTO, and CPSO had a large amount of ω-6 linoleic acid (C18:2 > 50%). Additionally, the fatty acid profiles of CPOs affected their nutritional quality and human health. The calculated AI, TI, and HH indexes can help in the evaluation of the risk of chronic diseases and metabolic syndromes. The CPRO, CPCO, CPSO, and CPLO revealed low AI and TI but a high HH ratio. In fact, the consumption of these oils can be associated with a reduced risk of several chronic diseases. On the other hand, the highest PUFA level in CPLO decreased its oxidative stability, which resulted in the lowest IP value (4.87 h). However, high amounts of antioxidants in the studied CPOs increased their AA and oxidation stability. Furthermore, the highest TSC (684 mg/100 g) in CPRO enhanced its health-promoting properties. Most importantly, concentrations of hazardous oxidation products and toxic PAHs in the investigated CPOs were below the legal limits recommended by EU food legislation and Codex Alimentarius standards.

In addition, the chemometric analyses clarified the interactions between the physicochemical parameters of the studied CPOs, their sensory attributes, and consumer acceptance. The bitter taste, astringency, and herbs-like flavour were negative descriptors, while the consumers favoured the sweet taste and roasted flavour. Based on sensory analysis, the CPPO showed the highest level of consumer preference, whereas the sensory attributes of CPLO had a negative impact on consumer attitude and purchase behaviour.

## Figures and Tables

**Table 1:** Fatty acid compositions and nutritional values of the studied cold-pressed oils.

Fatty Acid (%)	Oil Sample					
	CPLO	CPPO	CPMTO	CPRO	CPCO	CPSO
C16:0	5.93 ± 0.19 ^c^	12.41 ± 0.13 ^f^	8.37 ± 0.08 ^e^	4.63 ± 0.03 ^a^	5.41 ± 0.13 ^b^	6.58 ± 0.06 ^d^
C18:0	5.44 ± 0.19 ^d^	5.90 ± 0.11 ^e^	5.45 ± 0.21 ^d^	1.61 ± 0.06 ^a^	2.38 ± 0.07 ^b^	3.52 ± 0.17 ^c^
C20:0	0.22 ± 0.01 ^a^	0.41 ± 0.01 ^b^	2.90 ± 0.04 ^e^	0.57 ± 0.01 ^c^	1.62 ± 0.02 ^d^	0.20 ± 0.00 ^a^
C22:0	0.04 ± 0.00 ^a^	0.10 ± 0.00 ^b,c^	1.91 ± 0.33 ^e^	0.31 ± 0.00 ^c^	0.31 ± 0.00 ^c^	0.70 ± 0.00 ^d^
C24:0	<DL	0.10 ± 0.00 ^a^	0.90 ± 0.00 ^c^	<DL	<DL	0.20 ± 0.00 ^b^
ΣSFA	11.63	18.92	19.53	7.12	9.72	11.20
C16:1	0.07 ± 0.00 ^a^	0.10 ± 0.00 ^b^	0.10 ± 0.00 ^b^	0.18 ± 0.01 ^c^	0.10 ± 0.00 ^b^	0.10 ± 0.00 ^b^
C18:1	19.85 ± 0.18 ^b^	30.40 ± 0.26 ^d^	24.69 ± 0.13 ^c^	63.26 ± 0.06 ^e^	18.11 ± 0.21 ^a^	30.64 ± 0.54 ^d^
C20:1	0.13 ± 0.00 ^a^	0.10 ± 0.00 ^a^	0.90 ± 0.00 ^b^	1.28 ± 0.02 ^c^	14.43 ± 0.19 ^d^	0.10 ± 0.00 ^a^
C22:1	<DL	0.10 ± 0.00 ^a^	<DL	0.11 ± 0.00 ^a^	2.69 ± 0.13 ^b^	<DL
ΣMUFA	20.05	30.70	25.69	64.83	35.33	30.84
C18:2	16.30 ± 0.11 ^a^	50.39 ± 0.36 ^d^	56.46 ± 0.06 ^e^	19.82 ± 0.09 ^c^	19.38 ± 0.20 ^b^	57.64 ± 0.08 ^f^
C18:3	52.12 ± 0.28 ^d^	0.20 ± 0.00 ^a^	0.30 ± 0.00 ^a^	8.12 ± 0.14 ^b^	30.29 ± 0.21 ^c^	0.10 ± 0.00 ^a^
ΣPUFA	68.42	50.59	56.76	27.94	49.67	57.74
COX	13.14	5.54	6.13	4.43	8.72	6.26
AI	0.06	0.08	0.07	0.02	0.03	0.04
TI	0.06	0.45	0.33	0.09	0.07	0.23
HH	14.79	6.47	9.62	19.53	12.30	13.23

Results are expressed as mean percentage of total fatty acids ± standard deviation (SD) (*n* = 3); different letters (a–f) within the same rows indicate significant differences between the percentages of fatty acids of cold-pressed oils (one-way ANOVA and Duncan test, *p* < 0.05). Abbreviations: DL—detection limit; C16:0—palmitic acid; C18:0—stearic acid; C20:0—arachidic acid; C22:0—behenic acid; C16:1—palmitoleic acid; C18:1—oleic acid; C20:1—eicosenoic acid; C18:2—linoleic acid; C18:3—linolenic acid; SFA—saturated fatty acids; MUFA—monounsaturated fatty acids; PUFA—polyunsaturated fatty acids; COX—calculated oxidisability value; AI—atherogenicity index; TI—thrombogenicity index; HH—ratio of hypocholesterolemic to hypercholesterolemic fatty acids; CPLO—cold-pressed linseed oil; CPPO—cold-pressed pumpkin oil; CPMTO—cold-pressed milk thistle oil; CPRO—cold-pressed rapeseed oil; CPCO—cold-pressed camelina oil; CPSO—cold-pressed sunflower oil.

**Table 2:** Tocopherol compositions and contents in the studied cold-pressed oils.

Tocopherol Content (mg/100 g)	Oil Sample					
	CPLO	CPPO	CPMTO	CPRO	CPCO	CPSO
α-Tocopherol	1.78 ± 0.02 ^a^	7.49 ± 0.34 ^b^	38.91 ± 0.67 ^d^	27.00 ± 1.18 ^c^	1.20 ± 0.04 ^a^	73.37 ± 2.61 ^e^
β-Tocopherol	<DL	<DL	2.84 ± 0.08 ^b^	<DL	<DL	2.56 ± 0.04 ^a^
γ-Tocopherol	42.26 ± 1.02 ^b^	56.96 ± 0.55 ^c^	4.34 ± 0.20 ^a^	42.11 ± 0.74 ^b^	74.27 ± 1.85 ^d^	<DL
δ-Tocopherol	<DL	<DL	<DL	1.10 ± 0.05 ^a^	1.51 ± 0.03 ^b^	<DL
TTC	44.04 ± 1.04 ^a^	64.45 ± 0.50 ^b^	46.09 ± 0.57 ^a^	70.21 ± 1.04 ^c^	76.98 ± 1.78 ^d^	75.93 ± 2.57 ^d^

Results are expressed as mean ± standard deviation (SD) (*n* = 3); different letters (a–e) within the same rows indicate significant differences between tocopherol contents in cold-pressed oils (one-way ANOVA and Duncan test, *p* < 0.05). Abbreviations: DL—detection limit; TTC—total tocopherol content; CPLO—cold-pressed linseed oil; CPPO—cold-pressed pumpkin oil; CPMTO—cold-pressed milk thistle oil; CPRO—cold-pressed rapeseed oil; CPCO—cold-pressed camelina oil; CPSO—cold-pressed sunflower oil.

**Table 3:** Sterol compositions and contents in the studied cold-pressed oils.

Sterol (mg/100 g)	Oil Sample					
	CPLO	CPPO	CPMTO	CPRO	CPCO	CPSO
Cholesterol	1 ± 0 ^a,b^	1 ± 0 ^a,b^	46 ± 1 ^d^	2 ± 0 ^b^	26 ± 1 ^c^	<DL
Brassicasterol	3 ± 0 ^b^	<DL	1 ± 0 ^a,b^	73 ± 3 ^d^	22 ± 1 ^c^	<DL
∆-5-Avenasterol	44 ± 2 ^e^	8 ± 0 ^a^	10 ± 0 ^b^	10 ± 0 ^b^	35 ± 1 ^d^	15 ± 1 ^c^
β-Sitosterol	166 ± 5 ^a^	182 ± 10 ^b^	192 ± 3 ^b^	336 ± 19 ^e^	265 ± 5 ^d^	218 ± 4 ^c^
∆-7-Avenasterol	1 ± 0 ^a^	57 ± 2 ^d^	21 ± 1 ^c^	<DL	<DL	16 ± 1 ^b^
∆-7-Stigmasterol	5 ± 0 ^a^	21 ± 2 ^b^	141 ± 4 ^d^	2 ± 0 ^a^	4 ± 0 ^a^	61 ± 3 ^c^
Stigmasterol	27 ± 1 ^c^	2 ± 0 ^a^	37 ± 1 ^d^	3 ± 0 ^a^	9 ± 0 ^b^	26 ± 1 ^c^
Campesterol	80 ± 2 ^c^	7 ± 0 ^a^	30 ± 1 ^b^	249 ± 2 ^e^	110 ± 3 ^d^	32 ± 1 ^b^
Unidentified steroles	8 ± 1 ^a^	22 ± 1 ^d^	31 ± 1 ^e^	9 ± 0 ^b^	8 ± 1 ^a^	15 ± 1 ^c^
TSC	335 ± 7 ^b^	300 ± 10 ^a^	509 ± 4 ^e^	684 ± 13 ^f^	479 ± 6 ^d^	383 ± 6 ^c^

Results are expressed as mean ± standard deviation (SD) (*n* = 3); different letters (a–f) within the same rows indicate significant differences between sterol contents in cold-pressed oils (one-way ANOVA and Duncan test, *p* < 0.05). Abbreviations: DL—detection limit; TSC—total sterol content; CPLO—cold-pressed linseed oil; CPPO—cold-pressed pumpkin oil; CPMTO—cold-pressed milk thistle oil; CPRO—cold-pressed rapeseed oil; CPCO—cold-pressed camelina oil; CPSO—cold-pressed sunflower oil.

**Table 4:** Total phenolic content and antioxidant activity of the studied cold-pressed oils.

Oil Sample	Total Phenolic Content (mg GA/100 g)	Antioxidant Activity (μmol TE/100 g)		
	TPC	DPPH	ABTS	FRAP
CPLO	2.93 ± 0.20 ^a^	185.36 ± 7.62 ^a^	1040.86 ± 41.69 ^b^	78.63 ± 1.64 ^b^
CPPO	8.32 ± 0.12 ^e^	396.63 ± 12.69 ^d^	1638.58 ± 16.94 ^d^	119.21 ± 3.49 ^d^
CPMTO	5.42 ± 0.12 ^d^	234.65 ± 9.85 ^b^	958.59 ± 44.52 ^a^	61.93 ± 2.56 ^a^
CPRO	4.93 ± 0.11 ^c^	293.10 ± 10.67 ^c^	1328.00 ± 59.57 ^c^	99.67 ± 1.48 ^c^
CPCO	4.17 ± 0.23 ^b^	396.04 ± 11.45 ^d^	1367.50 ± 16.94 ^c^	75.80 ± 1.95 ^b^
CPSO	5.25 ± 0.15 ^c,d^	241.06 ± 12.86 ^b^	1085.10 ± 17.83 ^b^	62.22 ± 2.59 ^a^

Results are expressed as mean ± standard deviation (SD) (*n* = 3); different letters (a–e) within the same columns indicate significant differences between total phenolic content (TPC) and antioxidant activity of cold-pressed oils (one-way ANOVA and Duncan test, *p* < 0.05). Abbreviations: DPPH—2,2-diphenyl-1-picrylhydrazyl method; ABTS—2,2′-azino-bis(3-ethylbenzothiazoline-6-sulfonic acid); FRAP—ferric reducing antioxidant power; CPLO—cold-pressed linseed oil; CPPO—cold-pressed pumpkin oil; CPMTO—cold-pressed milk thistle oil; CPRO—cold-pressed rapeseed oil; CPCO—cold-pressed camelina oil; CPSO—cold-pressed sunflower oil; GA—gallic acid; TE—Trolox equivalent.

**Table 5:** Oxidative stability and quality parameters of the studied cold-pressed oils.

Parameter	Oil Sample					
	CPLO	CPPO	CPMTO	CPRO	CPCO	CPSO
IP (h)	4.87 ± 0.21 ^a^	9.47 ± 0.25 ^c^	9.03 ± 0.42 ^c^	12.93 ± 0.15 ^d^	5.37 ± 0.23 ^b^	9.23 ± 0.25 ^c^
PV (meq O_2_/kg)	0.61 ± 0.02 ^c^	2.44 ± 0.06 ^d^	2.88 ± 0.14 ^e^	0.42 ± 0.02 ^b^	0.24 ± 0.01 ^a^	4.61 ± 0.11 ^f^
pAnV (-)	0.39 ± 0.02 ^a^	4.77 ± 0.09 ^f^	0.66 ± 0.07 ^b^	0.88 ± 0.06 ^d^	1.88 ± 0.16 ^e^	0.79 ± 0.07 ^c^
TOTOX	1.61	9.65	6.42	1.72	2.36	10.01
AV (mg KOH/g)	0.37 ± 0.01 ^a^	1.55 ± 0.02 ^d^	2.83 ± 0.03 ^e^	0.42 ± 0.02 ^b^	0.31 ± 0.08 ^a^	1.16 ± 0.02 ^c^
FFA (%)	0.18 ± 0.01 ^a^	0.77 ± 0.01 ^d^	1.42 ± 0.02 ^e^	0.23 ± 0.01 ^b^	0.15 ± 0.04 ^a^	0.58 ± 0.01 ^c^
WVC (%)	0.085 ± 0.002 ^e^	0.030 ± 0.000 ^b^	0.090 ± 0.000 ^f^	0.045 ± 0.001 ^c^	0.059 ± 0.002 ^d^	0.020 ± 0.000 ^a^
B(a)P (µg/kg)	0.41 ± 0.01 ^c^	0.82 ± 0.02 ^e^	0.32 ± 0.01 ^b^	0.81 ± 0.02 ^e^	0.20 ± 0.01 ^a^	0.48 ± 0.02 ^d^
Chry (µg/kg)	0.76 ± 0.02 ^d^	0.54 ± 0.03 ^c^	0.83 ± 0.03 ^e^	0.28 ± 0.01 ^b^	0.21 ± 0.01 ^a^	0.54 ± 0.03 ^c^
B(a)A (µg/kg)	7.16 ± 0.05 ^f^	0.84 ± 0.01 ^c^	0.75 ± 0.03 ^b^	2.12 ± 0.03 ^e^	0.51 ± 0.02 ^a^	1.28 ± 0.06 ^d^
B(b)F (µg/kg	0.43 ± 0.02 ^d^	0.19 ± 0.01 ^a^	0.22 ± 0.01 ^b^	0.60 ± 0.02 ^e^	0.24 ± 0.01 ^b^	0.28 ± 0.01 ^c^
∑4PAHs (µg/kg)	8.76	2.39	2.12	3.81	1.16	2.58

Results are expressed as mean ± standard deviation (SD) (*n* = 3); different letters (a–f) within the same rows indicate significant differences between oxidative stability and quality parameters of cold-pressed oils (one-way ANOVA and Duncan test, *p* < 0.05). Abbreviations: IP—induction period; PV—peroxide value; pAnV—anisidine value; TOTOX—total oxidation value; AV—acid value; FFA—free fatty acids; WVC—water and volatile matter content; B(a)P—benzo(a)pyrene; Chry—chrysene; B(a)A—benzo(a)anthracene; B(b)F—benzo(b)fluoranthene; Σ4PAHs—sum of four specific polycyclic aromatic hydrocarbons; CPLO—cold-pressed linseed oil; CPPO—cold-pressed pumpkin oil; CPMTO—cold-pressed milk thistle oil; CPRO—cold-pressed rapeseed oil; CPCO—cold-pressed camelina oil; CPSO—cold-pressed sunflower oil.

**Table 6:** Sensory attributes for the studied cold-pressed oils.

Sensory Attributes	Description
OFI	The intensity of all flavour and taste attributes taken together
Sweet taste	The basic taste simulated by sugar
Bitter taste	The basic taste elicited by quinine and caffeine
Herbs-like flavour	The flavour reminiscent of herbs
Cabbage-like flavour	The flavour associated with asparagus, cabbage, or fresh green vegetables
Seed-like flavour	The flavour associated with fresh seeds
Mustard-like flavour	The flavour associated with mustard, onion, and spiciness
Nutty flavour	The flavour associated with fresh nuts
Roasted flavour	The flavour associated with roasted oils
Wood-like flavour	The flavour associated with fresh, dry, cut wood
Medicine-like flavour	The flavour reminiscent of medicine, hospital, and pharmacy
Persistence	How long do flavour sensations remain as aftertaste
Astringency	The shrinking or drying effect on the tongue surface elicited by tannins

## Data Availability

The data presented in this study are available on request from the corresponding author.

## Sample Availability

Samples of the compounds are available from the authors.
